# Threshold Effect in the Relationship between Environmental Regulations and Haze Pollution: Empirical Evidence from PSTR Estimation

**DOI:** 10.3390/ijerph182312423

**Published:** 2021-11-25

**Authors:** Yonglian Chang, Yingjun Huang, Manman Li, Zhengmin Duan

**Affiliations:** 1 School of Economics and Business Administration, Chongqing University, Chongqing 400030, China; Huangyj@cqu.edu.cn; 2 Department of Statistics and Actuarial Science, College of Mathematics and Statistics, Chongqing University, Chongqing 400030, China; lmm@cqu.edu.cn (M.L.); dzm@cqu.edu.cn (Z.D.)

**Keywords:** direct threshold effect, indirect threshold effect, environmental regulation, non-linear, haze pollution, PSTR

## Abstract

The impact of environmental regulations (ER) on haze pollution control has been continuously debated in the field of sustainable development. This paper explores the direct and indirect threshold effects of ER on haze pollution, and five underlying mechanisms—technological innovation (TI), industrial structure (IS), foreign direct investment (FDI), urbanization (UR), and electricity consumption (EC)—are adopted to investigate the indirect threshold effects. Panel data, over the period 2008–2018, of 284 Chinese cities were used and the threshold effects were predicted endogenously based on the panel smooth transition regression (PSTR) model. The results showed the following: (1) For the direct threshold effect, there exists a U-shaped relationship between ER and haze pollution. ER significantly reduced haze pollution when ER < 38.86 due to “cost effects”. However, ER increased haze pollution after the threshold owing to the “green paradox”, which was not significant. (2) For the indirect threshold effect, when TI = 0.37, IS = 39.61, FDI = 7.25, and UR = 42.86, the relationships between ER and haze pollution changed. The changes and corresponding reasons for the indirect threshold effects are discussed in detail. (3) After a comprehensive analysis, the threshold effects have obvious regional distribution characteristics and internal connections. Finally, based on the results, it is essential for governments to enact appropriate environmental regulatory policies and enhance inter-regional synergies in environmental governance.

## 1. Introduction

**Figure 1:**
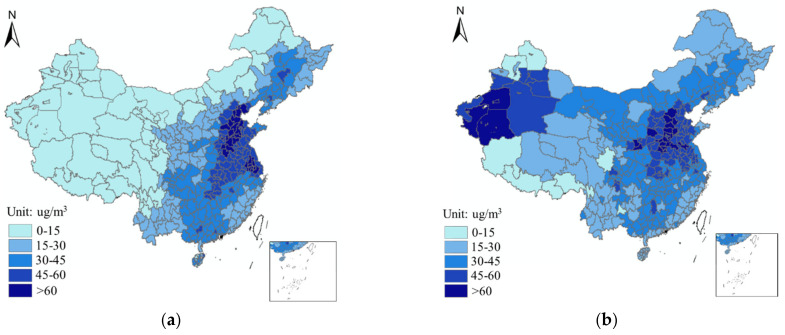
(**a**) PM_2.5_ concentration (μg/m^3^) of China in 2009; (**b**) PM_2.5_ concentration (μg/m^3^) of China in 2018.

China’s economy and urbanization have developed rapidly since the Government implemented the “Chinese Reform and Opening up Policy”, leading to severe environmental problems, particularly haze pollution [1] as shown in Figure 1. According to China’s Eco-environmental Status Bulletin 2018 released by the Chinese Ministry of Ecology and Environment, in 338 prefecture-level cities the environment was evaluated, of which only 121 cities (35.8%) met the standard concentration of PM_2.5_ (35 μg/m^3^) with an annual average PM_2.5_ concentration of 39 ug/m^3^. Importantly, increasing environmental problems have drawn wide attention from all walks of life.

In recent years, the Chinese Government has issued some environmental regulations (ER) to alleviate haze pollution and achieve environmental protection [2]. In 2011, environmental protection was written into the “Chinese Twelfth Five-Year Plan”, moreover, the air quality of all prefecture-level cities should be monitored. In 2015, the “Environmental Protection Inspection Plan” was issued by the State Council. In 2020, the Central Office of the Chinese Communist Party (CPC) and the State Council jointly issued “Guiding Opinions on Building Modern Environmental Governance System”. Under the call of national environmental protection, the local government also formulated relevant environmental policies to control air pollution. Although the Government has devoted itself to improving the environment through the above environmental regulations, haze pollution in China continues to worsen (Figure 1). This study attempts to uncover and provide answers regarding the effectiveness of China’s environmental regulations on haze pollution.

In the existing literature, Cole et al. [3], Blackman and Kildegaard [4] and, Zhang et al. [5] analyzed the direct relationship between environmental regulations (ER) and pollution, including the linearity and nonlinearity, and obtained different results [6]. The linear relationship means that ER have a single positive or negative effect on the environment, and this relationship has been investigated by many researchers. The GMM (generalized method of moments) estimation method was used by Hao et al. [7] who confirmed that ER have a positive impact on the performance of the environment, a conclusion that was also reached by Cole et al. [3], that regulations, both formal and informal, were effective to mitigate pollution intensity. On the contrary, Blackman and Kildegaard [4] postulated that strict ER will not increase the use of clean technologies and improve environmental pollution. Zhang et al. [5] stated that government regulations have negative impacts on environmental performance. Besides the linear relationship, few scholars began investigating the nonlinear relation between the ER and the performance of the environment. In general, the impact of environmental supervision on green growth is non-linear [8]—both formal and informal ER are important to mitigate the pollution—and the strength of these regulations is different at different stages [9], and in most cases, different environmental supervision tools are always implemented to reduce haze pollution [8]. A U-shaped relationship exists, in which regulations first play a positive effect and then have an inverse impact after the inflection point [10]. However, Zhou et al. [11] found that there is an inverted U-shaped relationship between PM_2.5_ concentration and environmental regulations when adding the square term of the regulations.

Generally speaking, the indirect relationship between ER and haze pollution is investigated through mechanisms including technological innovation (TI), industrial structure (IS), foreign direct investment (FDI), urbanization (UR), electric power consumption (EC), and other mechanisms. Environmental regulations have two effects on technological innovation, including the positive compensation effect of the “Porter hypothesis” [12] and the negative offset effect known as the “compliance cost effect” [13]. China’s haze pollution is mainly caused by industrial activities due to the traditional industrial structure [14], which forces the Government to emphasize environmental regulations [15]. Meanwhile, the FDI also has a double-edged sword impact on the effectiveness of environmental regulations, known as the “pollution halo” effect [16] and “pollution haven” effect [17]. Thus, some scholars have integrated these factors to analyze the effectiveness of environmental regulations. Song et al. [2] investigated the indirect effects of environmental regulations by technological innovation and industrial structure, which is similar to the research of Zhang et al. [13] that explored the indirect effects through four underlying mechanisms—technological innovation, industrial structure, foreign direct investment, and coal consumption. In addition, Dogan and Turkekul [18] found that UR and EC will worsen the environment in the long term, results that are confirmed by Liang et al. [1]. Industrial pollutant emissions can be reduced by developing new energy sources to achieve energy-saving—and improve—environmental quality [19]. On this basis, this research uses five underlying mechanisms: technological innovation (TI), industrial structure (IS), foreign direct investment (FDI), urbanization (UR), and electricity consumption (EC).

Although other scholars (Zhang et al. [13], Dogan and Turkekul [18]) have contributed relevant research, the threshold effects regarding the direct and indirect relationship between environmental regulation and haze pollution have not been considered. Previous studies in the literature showed a linear relationship, especially for indirect relationships. Moreover, the spatial distribution characteristics of direct and indirect effects and their internal connections are also unclear. In addition, when investigating the linear and nonlinear impact of environmental regulations on haze pollution, most empirical studies have adopted simplified regression models that rely on the polynomial form of the quadratic function of the explanatory variables for testing [11,20,21]. However, these simplified models are not only improper for explaining the reason for the predicted relationship but are not suitable for explaining the estimated coefficient both structurally and economically [22]. Meanwhile, most previous research has focused on Chinese haze pollution at the provincial level, while minor attention has been given to prefecture-level cities [2].

This paper uses data from 284 Chinese cities (2008–2018 period) to explore the threshold effects of ER on PM_2.5_ as an indicator of haze pollution. The PSTR model is adopted to explore both direct and indirect threshold effects through five underlying mechanisms: technological innovation (TI), industrial structure (IS), foreign direct investment (FDI), urbanization (UR), and electricity consumption (EC). Finally, this article comprehensively analyzes the spatial distribution characteristics and internal relations of direct and indirect threshold effects. The contributions of this study include the following: (1) The threshold effects of the direct relationship of environmental regulations (ER) and haze pollution are investigated. (2) The threshold effects of the indirect ER on haze pollution are clarified. (3) The spatial distribution characteristics and internal relations of direct and indirect threshold effects are comprehensively analyzed. (4) The PSTR model is applied to determine the dynamics of ER, which can appropriately deal with major econometric problems [23]. (5) Using text analysis, a special environmental regulation variable is constructed to explore the haze control effect [24].

## 2. The Underlying Mechanisms

### 2.1. The Direct Effects Analysis

**Figure 2:**
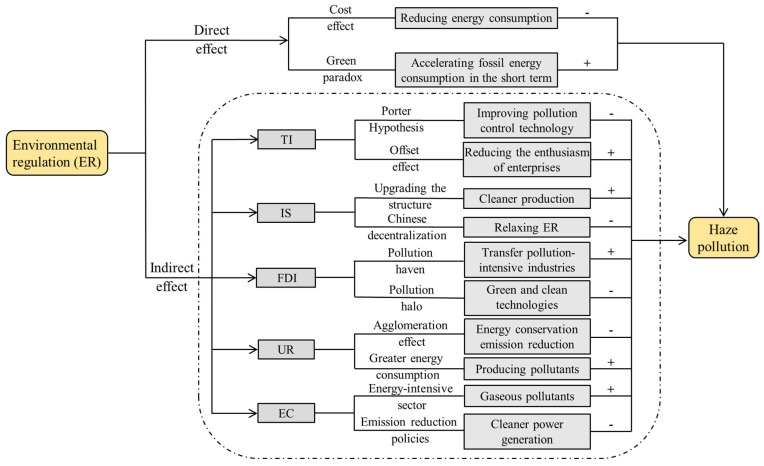
The impact paths of environmental regulation on haze pollution.

Existing research on the effectiveness of ER on haze pollution includes two perspectives. On the one hand, environmental regulations have significantly reduced haze pollution [7]. The reason is that enterprises must implement environmental protection measures under the premise of complying with the Government’s ER, in which the cost for pollution reduction will inevitably increase, known as the “cost effect” [2]. This reduces energy consumption, thereby achieving the goal of reduced haze pollution. Another view is the “green paradox”, proposed by Sinn [25], in which environmental supervision accelerates the consumption of fossil energy causing environmental degradation in the short term. In general, the “green paradox effect” and “cost effect” coexist—the former is weaker than the latter. Because traditional energy is easy to store, it will be consumed in the long term; moreover, the scale and cost of the enterprise are limited in the short term, thus, they will not increase energy demand and consumption [2,26]. The direct effects are shown in Figure 2.

### 2.2. Indirect Effects

Environmental regulations can indirectly control haze pollution by addressing industrial and human production activities that are the sources of haze pollution. Many researchers are devoted to exploring the indirect factors and have found that technological innovation (TI) [23,27], industrial structure (IS) [19,28], foreign direct investment (FDI) [16], urbanization (UR) [18], and the power consumption of electricity (EC) [29] are important factors affecting environmental pollution. The five underlying mechanisms are shown in Figure 2.

#### 2.2.1. The Effect of ER on Haze Pollution by TI

ER have two effects on technological innovation, including a positive compensation effect known as the “Porter hypothesis” [12] and a negative offset effect known as the “compliance cost effect”, which can further indirectly affect haze pollution. For the positive compensation effect, under the influence of environmental regulations, enterprises will expand capital investment in environmental protection technologies to improve green productivity and avoid administrative penalties, which will lead to the “compensation cost effect” [27,28]. However, owing to strict ER, enterprises must increase investment in pollution control in order to achieve cleaner production, which will, in turn, reduce investments in science and technology [24]. Moreover, the interest of enterprises to understand low-pollution technology will be reduced by the compliance cost effect. The above-mentioned reasons will impede technological innovation and are not beneficial to improving the air quality [30].

#### 2.2.2. The Effect of ER on Haze Pollution by IS

China’s haze pollution is mainly caused by industrial activities due to the traditional industrial model which sacrifices the environment in exchange for development. In response, the Government introduced a successive series of environmental regulations to solve environmental problems [15,31]. Environmental governance has become an indispensable—annual—part of government work, which promotes industrial structure (IS) upgrading by decreasing the number of heavily polluting industries, as a result, current production methods will be impacted [8]. However, owing to the “Chinese decentralization” system [32], the main basis for the higher-level government to evaluate the lower-level government is the GDP relative performance evaluation index, which will enable local governments to support enterprises for developing the local economy by relaxing ER [33]. The heavier the secondary industry, the more serious pollution is [11]. Therefore, this article uses the proportion of the secondary industry’s added value to represent the IS.

#### 2.2.3. Impact of ER on Haze Pollution by FDI

Over the past few decades, as a result of the Reform and Opening-up Policy, China has attracted a great deal of foreign direct investment (FDI). In theory, FDI has a double-edged sword effect on the environment in China. Polluting industries from developed countries flood into China under weak ER, placing a significant burden on the environment, subsequently turning China into a “pollution haven” [17,34]. On the contrary, FDI from developed countries will introduce cleaner technologies to developing countries. These technologies can achieve the goal of cleaner production and reduce the host country’s environmental pollution, known as the “pollution halo” hypothesis [35,36].

#### 2.2.4. Impact of ER on Haze Pollution by UR

Although China’s rapid urbanization progress has promoted its economic development, it has also accelerated the exploitation of limited resources and caused many environmental problems [37], such as consuming large amounts of fossil fuels and the production of exhaust gas, wastewater, solid waste, etc. [38]. However, due to the scale effect and agglomeration effect, urbanization not only achieves intensive development but also helps enterprises and residents achieve green development. Therefore, this will be more conducive to exerting a restraint effect of ER and achieving emissions reduction [39,40]. Overall, different stages of urbanization will have varying impacts on environmental pollution [41,42].

#### 2.2.5. Impact of ER on Haze Pollution by EC

In general, in the past years, traditional thermal power industries in China have provided energy requirements for the most productive and economic activities, thus these industries are indispensable for social development [43,44]. However, a range of environmental pollutants from these industries are also emitted during electricity production processes [29]. Thus, a number of energy-saving and emissions-reducing policies have been released by the Government, aimed at promoting the low-carbon development of the power industries. Accompanied by the promulgation of these policies, clean power generating methods have also grown rapidly, such as hydropower and wind power technology [45], which are more environmentally friendly. The impact of ER on haze pollution through power consumption will be discussed in detail in the following section.

## 3. Data Description and Model Settings

### 3.1. Data and Variables

Although province-level governments usually promulgate programmatic policies, these policies are implemented by lower-level governments (prefecture-level cities) under their jurisdiction. Therefore, in the present study, the panel data (over the period 2008–2018) of 284 Chinese cities are adopted. The variables are collected from *“City Statistical Yearbook in China”* (2009–2019), “*Work Reports and Statistical Yearbooks of Province-level Government*” of 30 provinces, and the “*Statistical Bulletin on Social and Economic Development”* of each city. Among them, PM_2.5_ was selected as the dependent variable, and the original PM_2.5_ data were obtained from the PM_2.5_ density database, established by Columbia University.

In order to avoid heteroscedasticity, some indicators adopt logarithmic processing. Missing data in some cities are supplemented by the average growth rate method; moreover, some cities that have substantial missing data are eliminated. Table 1 shows the concrete information of the selected variables.

When using the PSTR model, transition variables are involved to observe the threshold effect of environmental regulations on haze pollution. The transition variables generally select important exogenous variables that have an influence on the nonlinear relationship obtained from the perspective of economic theory [52]. This article selects five transition variables that are analyzed in Section 2.

**Figure 3:**
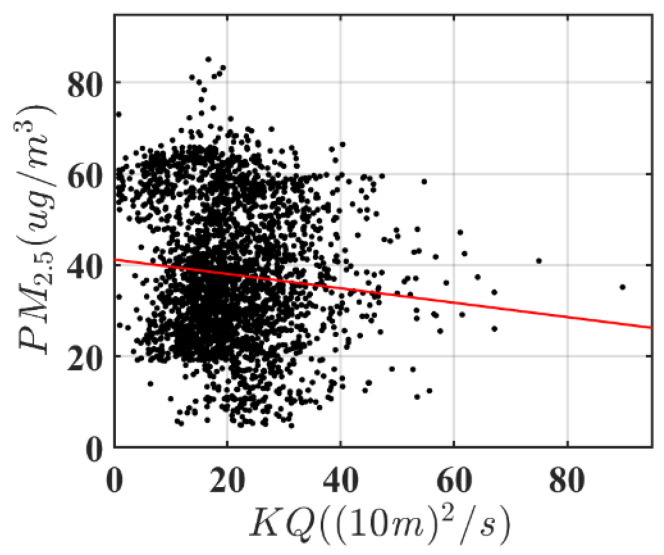
Scatter diagram and regression line of the correlation between PM_2.5_ and air flow coefficient (KQ).

Haze pollution has a strong “spatial spillover effect”; therefore, the air flow coefficient (*KQ*) is used according to previous research by Chen and Chen [14] to control the spillover effect. The *KQ* equation is expressed as follows:

(1) $$KQ_{ij}=WS_{ij}\times BLH_{ij}$$

where *i* and *j* denote the city and year, respectively. *KQ* represents the air flow coefficient, *WS* denotes the average wind speed, and *BLH* is the atmospheric boundary layer height. *WS* and *BLH* are determined by complex meteorological systems and geographical conditions, and satisfy the exogenous assumption of effective instrumental variables [51]. Figure 3 shows a significantly negative relationship between the *KQ* and PM_2.5_ concentration.

### 3.2. Model Settings

This study adopts a PSTR model (proposed by González et al. [53]) to improve the interpretation of the observed U-shaped (or inverted U-shaped) relationship. The PSTR model has some merits: (1) It can properly evaluate sharp and smooth transitions between regimes, which is different from previous non-linear models that assume an abrupt switch from one regime to another (such as, Markov transition model and panel threshold); (2) it can deal with major econometrics issues, including heterogeneity and temporal problem, through smooth changes in variables [23]; (3) in recent years, previous studies have verified that this model can appropriately evaluate the non-linear relationship between financial and economic variables [54,55].

A simple case is assumed herein to clearly illustrate the PSTR model, which has two regimes and a transition function.

(2) $$Y_{it}=\alpha_{i}+\beta_{0}X_{it}+\beta_{1}X_{it}*G(q_{it};\gamma ,c)+\varepsilon_{it}$$

where *i* = 1,…, *N* represents the number of cities in the panel data, and *t* = 1,…, *N* is the time dimension. *α_i_* is the fixed effect factor (unobserved heterogeneous intercept). The endogenous variable *Y_it_* represents the annual average PM_2.5_ concentration of selected cities in China, which is a matrix scalar. *X_it_* is a series of endogenous variables including the independent variable environmental regulations (ER) and other control variables such as PD, PGDP, PB, RP, and KQ. *ε_it_* is an error term. G(*q_it_*;*γ,c*) is the transition function of *q_it_*, and *q_it_* in this study represents the transition variables, which, in this research, includes technological innovation (TI), industrial structure (IS), foreign direct investment (FDI), urbanization (UR), and electric power consumption (EC). According to Colletaz et al. [56], Equation (3) denotes the concrete transition function:

(3) $$G(q_{it};\gamma ,c)={\left[1+\exp(-\gamma \prod_{j=1}^{m}\left(q_{it}-c_{j}\right))\right]}^{-1}$$

where *γ* represents the smoothness parameter, which is used to affect the transition speed of the transition function. *c_j_* is the position vector, *j* = 1...*m*, *m* denotes the dimension. The value range of the transition function is 0~1, which means there are two key stages in PSTR. When G(*q*_it_;*γ,c*) equals 0, the relationship between ER and haze pollution is defined by *β*_0_ in the first regime. If G(*q*_it_;*γ,c*) equals 1, the impact of ER on haze pollution is equal to *β*_0_ + *β*_1_ in the second regime. If *q*_it_ becomes larger, Equation (3) changes between *β*_0_ and *β*_0_ + *β*_1_. For instance, the elasticity of ER to haze pollution becomes:

(4) $$\frac{\partial Y_{it}}{\partial X_{it}}=\beta_{0}+\beta_{1}G(q_{it};\gamma ,c)$$

As γ→0, the PSTR becomes a linear fixed effects estimation; as γ→∞, the model turns into the PTR (panel threshold regression) model proposed by Hansen [57]. When the regimes are more than two in the PSTR model, the variable *Y_it_* can be expressed in Equation (5):

(5) $$Y_{it}=\alpha_{i}+\beta_{0}X_{it}+\sum_{j=1}^{r}\beta_{j}X_{it}*G_{j}(q_{it}^{j};\gamma ,c_{j})+\varepsilon_{it}$$

In Equation (5), *r* presents the transition function’s number in Equation (3). If “*r* = 0”, PSTR is the linear model, while when “*r* = 1 or 2”, two regimes or three regimes are considered, respectively.

A detailed process of the PSTR model is as follows:

(1) Linear (H_0_) and nonlinear testing (H_1_) is the first step before specifying and estimating a nonlinear model. The following Equations (6)–(8) are used for the linearity test [56]. The statistics are defined as follows:
  (6) $$LM_{w}=\frac{TN(SSR_{0}-SSR_{1})}{SSR_{0}}$$
  (7) $$LM_{F}=\frac{TN(SSR_{0}-SSR_{1})/mk}{SSR_{0}/(TN-N-mk)}$$
  (8) $$LRT=-2[\log(SSR_{1})-\log(SSR_{0})]$$

In Equations (6)–(8), *SSR*_0_ and *SSR*_1_ are the sums of squared panel residuals under different assumptions, *k* is the explanatory variable’s number. If *H*_0_ is not rejected, the model is a linear panel estimation, else, the number of transition functions and extreme regimes can be determined by *r*.

(2) The test H_0_: *r* = 1 against H_1_: *r* = 2 is assessed when the null hypothesis is not accepted in the first step. Then, repeat the process for H_0_: *r* = i against H_1_: *r* = i + 1, until H_0_ can be accepted [58].
(3) The final stage of PSTR analysis is the estimation stage. We refer to González et al. [53] to use nonlinear least squares (NLS) to estimate the model.

## 4. Results

### 4.1. Quantification of Environmental Regulations (ER)

Based on the research of Li and Ye [46] and Chen and Chen [14], first, the present study uses the text segmentation method [59] to obtain the proportion of environment-related vocabularies in the provincial government’s work report, which is aimed at controlling the heterogeneity that is caused by the length of the report. Then, referring to Bartik [47], multiply the proportion with IS to obtain the ER of the prefecture-level government. The summarized Equation (9) is as follows:

(9) $$ER_{ij}=\frac{EV_{ij}}{TV_{ij}}\times IS_{ij}$$

where *i* and *j* denote the city and year, respectively. *ER* represents the environmental regulations, *EV* denotes the sum of environment-related words in the work report of the provincial government where the city *i* is included. *TV* represents the sum of all vocabularies frequencies of the entire report, and *IS* means that the secondary industry’s added value accounts for the regional GDP.

The relevant vocabularies mainly include: “I (*PM_2.5_/PM_10_*), II (*CO_2_/SO_2_*), III (*atmosphere/air*), IV (*low carbon*), V (*ecology*), VI (*environmental protection/environmental improvement*), VII (*pollution*), VIII (*energy consumption*), IX (*emission reduction*), X (*green*), XI (*COD/sewage*)”. Table 2 shows the results of the vocabulary statistics.

**Figure 4:**
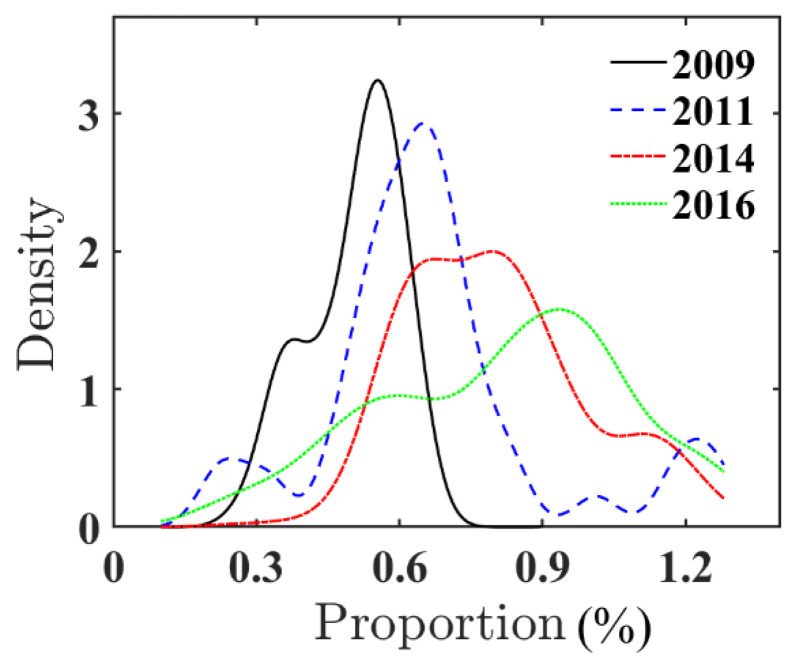
The proportion of environment-related words in the Government work report.

Figure 4 shows the nuclear density curve of the proportion of environment-related vocabularies in the Government’s work report. The proportion continues to increase over time, signifying that the Government is gradually paying attention to environmental problems.

### 4.2. Panel Unit Root Test

The unit root test assesses the presence of a unit root in a series, as the presence of a unit root leads to a non-stationary series, which further leads to a pseudo-regression in the regression analysis. Therefore, it is necessary to conduct the test. The unit root test is usually performed using the LLC (Levin–Lin–Chu) and Fisher-ADF test. The results of the LLC and Fisher-ADF test [60,61] are shown in Table 3. The null hypothesis—that the series has a unit root—is strongly rejected, which shows that all variables are stationary.

### 4.3. Results of Linearity Test

After determining that all variables are stationary, the first step of the PSTR model is to test linearity and nonlinearity. From the linear test results of *LM_W_*, *LM_F_*, and *LRT* reported in Table 4, all models reject the null hypothesis. Therefore, the models have at least one nonlinear threshold effect.

### 4.4. Results of Remaining No Linearity

At this stage, the *LM_W_*, *LM*_F_, and *LRT* tests will be repeated to determine the appropriate number of regimes [62]. The results are depicted in Table 5. At the conventional significance level of all the models, the *H*_0_ hypothesis cannot be rejected. Therefore, all models have a threshold effect that can be estimated using the two-regime PSTR model.

### 4.5. PSTR Estimation Results

#### 4.5.1. Analysis of Direct Effects

**Figure 5:**
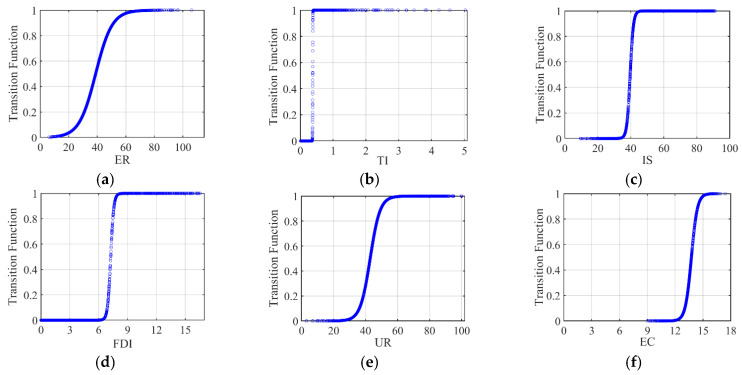
(**a**) Estimated transition function of ER; (**b**) estimated transition function of TI; (**c**) estimated transition function of IS; (**d**) estimated transition function of FDI; (**e**) estimated transition function of UR; (**f**) estimated transition function of EC.

**Figure 6:**
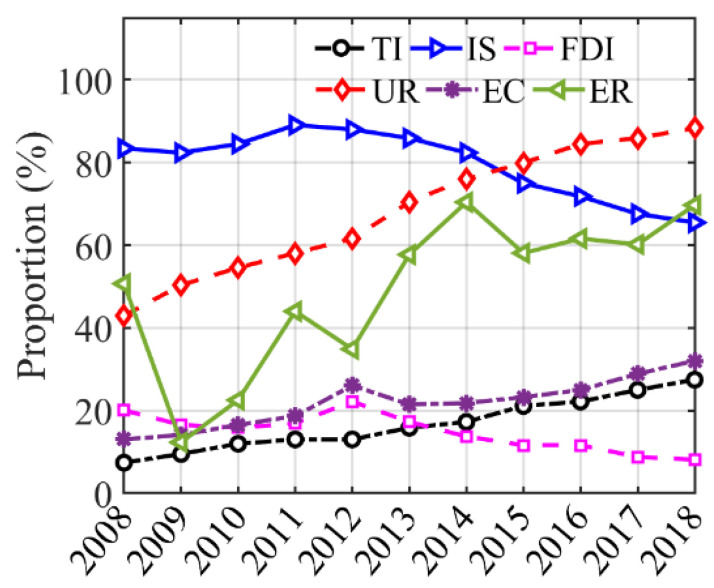
Trend graph of the proportion of cities over the threshold.

As shown in the first column of Table 6, there are two regimes in the impact of ER on haze pollution with a smooth slope parameter of 0.17 (Figure 5a). In the first regime (ER < 38.86), ER have a significant haze reduction effect and the coefficient is −0.06, indicating that ER have a significant restraining effect on the pollution behavior of enterprises due to the “cost effect”. On the contrary, in the second regime (ER > 38.86), ER increased the haze pollution with a coefficient of 0.02 (*β*_0_ + *β*_1_), which may be related to the short-term increase in energy consumption and caused environmental pollution due to the “green paradox” effect. However, the relationship in the second regime is not significant. Therefore, China’s ER have a significant haze reduction effect at the research stage; however, their role in promoting haze pollution has started to emerge. As shown in Figure 6, the proportion of cities that exceed the ER threshold illustrates a fluctuating upward trend. The Government should promptly adjust environmental regulations to prevent this ER promotion from becoming more significant.

#### 4.5.2. Analysis of Indirect Effects

The indirect effects results are also shown in Table 6. In the first regimes of the TI, IS, FDI, and UR models, the estimated coefficient of ER (*β*_0_) is statistically significant and negative, with values of −0.05, −0.001, −0.020, and −0.16, respectively. Moreover, it is found that this coefficient is positive in the EC model (1.22). In the second regime, the coefficients expressed as *β*_0_ + *β*_1_ are positive in the models other than the FDI and EC models (coefficients for TI, IS, and UR are 0.14, 0.001, and 0.04, respectively). The coefficient is found to be negative in the FDI model (−0.10) and EC model (−0.33). The effectiveness of environmental regulations on haze pollution varies with different transition variables and is analyzed in the following.

For the TI model results shown in Table 6, the transition changes sharply between regimes (Figure 5b) and the slope is 493.08. The results show that the increase in ER reduce the haze pollution with a coefficient of −0.05, before reaching the TI threshold (TI = 0.37). The advancement of environmental protection technology improves enterprises’ competitiveness and compensates for “compliance cost”, which is conducive to the treatment of haze pollution. The proportion of cities in Figure 6 that were over the threshold did not exceed 30%, which demonstrates that China’s current scientific and technological development is generally aimed at reducing haze pollution (Figure 6). However, the coefficient changes to 0.14 (*β*_0_ + *β*_1_) after the threshold. However, the large investment in science and technology—due to the offset effect and the compliance cost effect ER—has not achieved the expected effects. The proportion of cities that were over the threshold increased during the period 2008–2018, indicating that the offset effect of ER was gradually obvious (Figure 6).

For the IS model results shown in Table 6, the transition changes smoothly between regimes (Figure 5c) and the slope is 1.01. ER reduce haze pollution with a coefficient of −0.001 before the IS threshold (IS = 39.61). The reason is the difficulty for pollution-intensive industries to remain viable due to high “environmental compliance costs”. The proportion of cities that were over the threshold decreased during the period 2008–2018 as shown in Figure 6, which indicates that ER are effective in promoting the optimization of industrial structures. After the threshold, ER increased haze pollution with a coefficient of 0.001. Moreover, China still has a relatively high proportion (larger than 50%) of secondary industries (Figure 6). The “Chinese decentralization” system encourages local governments to ease ER and support enterprises in developing the local economy, which subsequently exacerbates haze pollution [32].

For the FDI model results shown in Table 6, the transition changes smoothly between regimes (Figure 5d) and the slope is 6.24 as shown in Table 6. The results show that ER reduce haze pollution with a coefficient of −0.02 when FDI < 7.25. A negative correlation is more obvious and the coefficient changes to −0.10 after the threshold. Moreover, it can be seen that FDI has become an important factor in reducing haze pollution. A substantial number of high-polluting foreign companies are forced to leave owing to strict ER [13], which can help China to avoid becoming a “pollution haven”. Moreover, FDI brings environmental protection technologies that mitigate the host country’s pollution [63]. Finally, according to Figure 6, the proportion of cities that are over the threshold gradually decreases and is under 25% during the period 2008–2018, indicating that ER prevent China from becoming a “pollution haven”; meanwhile, the number of foreign companies that can introduce green and cleaner production technologies is reduced [63].

For the UR model results shown in Table 6, the transition changes smoothly between regimes (Figure 5e) and the slope is 0.32. The results in Table 6 show that ER decreased the haze pollution with a coefficient of −0.16 before the UR threshold (UR = 42.86), and the proportion of cities that were over the threshold increased during the period 2008–2018 (Figure 6). The scale effect and agglomeration effect of UR are conducive to exerting the restraint effect of ER and achieving emissions reduction [39]. However, the coefficient changes to 0.04 when the UR larger is than 42.86, and the proportion is larger than 50% except for 2008 (Figure 6). Due to a high urbanization rate, the excessive consumption of energy will result in producing pollutants and reducing the binding effect of ER. Therefore, urbanization will indirectly aggravate environmental pollution.

For the EC model results shown in Table 6, the results show that transition changes smoothly between regimes (Figure 5f) and the slope is 3.10. When EC < 13.75, the ER exacerbates haze pollution with a coefficient of 1.22 in the regime. China’s power generation is dominated by coal, and the power industry provides the energy required for the most productive and economic activities, resulting in minimal environmental regulations efficiency. Furthermore, there is a significant negative effect (−0.33) of ER on haze pollution after the threshold. When power consumption is large, more pollutants are bound to be produced. Therefore, the Chinese Government has issued policies aimed at constructing a resource-saving society in regard to the power industry [64,65]. Moreover, environmentally friendly energy generating methods have been exploited. These measures can lead to effective environmental regulations with favorable external conditions, thus resulting in a haze reduction effect. According to Figure 6, the proportion of cities that were over the threshold increased during the period 2008–2018, indicating that ER and cleaner power generation are effective for improving the environmental quality. However, the proportion does not exceed 35%, which demonstrates that ER, in over half of the samples did, not achieve green development of the power industry. The power industry continues to exacerbate haze pollution.

#### 4.5.3. Comprehensive Analysis

**Figure 7:**
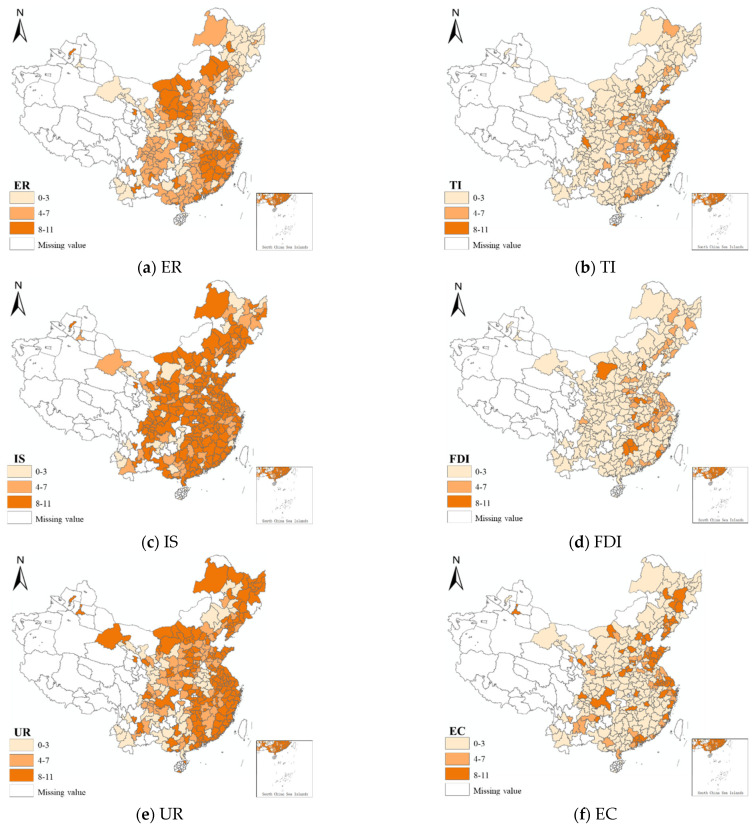
(**a**) The cumulative frequency of ER in 284 cities over the threshold during 2008–2018; (**b**) the cumulative frequency of TI in 284 cities over the threshold during 2008–2018; (**c**) the cumulative frequency of IS in 284 cities over the threshold during 2008–2018; (**d**) the cumulative frequency of FDI in 284 cities over the threshold during 2008–2018; (**e**) the cumulative frequency of UR in 284 cities over the threshold during 2008–2018; (**f**) the cumulative frequency of EC in 284 cities over the threshold during 2008–2018.

According to the results in Table 6, ER exacerbates haze pollution when ER, TI, IS, and UR are over the threshold. However, when the FDI and EC are over the threshold, ER reduce haze pollution. In order to investigate the regional distribution features of the threshold effect, the cumulative frequency of 284 cities that exceeded the threshold in all models during 2008–2018 was calculated and the spatial distribution is shown in Figure 7. Haze pollution in adjacent areas has spatial correlation characteristics [66], and the Chinese Government has emphasized joint pollution prevention [24]. Therefore, the ER in adjacent areas is similar, as shown in Figure 7a, and there are obvious contiguous areas in the governance effect of ER. From the distribution in Figure 7b, the areas with a higher frequency of TI are distributed in the eastern coastal areas, which are developed regions in China, thus, a more serious offset effect of TI in the eastern coastal area is observed. Due to China’s traditional development model, the industry has developed rapidly [2]. As shown in Figure 7c, all samples advance towards a higher frequency of IS, which will lead to the aggravation of haze pollution, and be a disadvantage to the governance. The distribution characteristics of FDI and TI are similar (Figure 7d)—a deeper influence of the opening-up policy in developed areas [67]. China is currently undergoing rapid urbanization of its population, especially in developed areas, and the population generally flows to industrial or economically developed areas. The distribution of UR is mainly in plains and coastal areas (Figure 7e), which is consistent with Liang et al. [68]. The high frequency of EC is distributed in coastal areas (Figure 7f), which benefits from the abundant wind energy in the region [69]. The regional distribution of TI, IS, and UR is similar to ER; ER will increase haze pollution after exceeding the corresponding threshold. There are fewer cities with high frequencies of FDI and EC, which cannot achieve a large-scale haze reduction effect. Therefore, the interaction of these indirect effects has caused a direct impact of ER on haze control.

## 5. Conclusions and Policy Recommendations

### 5.1. Conclusions

In this study, the direct and indirect threshold effects of ER on haze pollution were analyzed by using prefecture-level city panel data (2008–2018) in China. We explored the indirect threshold effect through five underlying mechanisms: technological innovation (TI), industrial structure (IS), foreign direct investment (FDI), urbanization (UR), and electricity consumption (EC). Finally, the inner connection between the two effects was explored. The PSTR model was used to overcome the problems of heterogeneity, endogeneity, and nonlinearity when investigating the impact of ER on haze reduction. This study provides new evidence for the effect of ER on haze control. The current study’s conclusions are as follows:

For the direct threshold effect, a U-shaped relationship was observed between ER and haze pollution. Due to “cost effects”, ER significantly reduced haze pollution when ER < 38.86. Moreover, there exists a “green paradox”, where ER increased haze pollution after the threshold; however, this relationship was not significant. Therefore, ER significantly reduced haze pollution in the research phase.

For the indirect threshold effect, U-shaped relationships were observed between ER and haze pollution through the TI, IS, and UR underlying mechanisms. ER effectively reduced the haze pollution before reaching the threshold, while the effectiveness of ER on haze pollution became inverse after the threshold (TI > 0.37, IS > 39.61, UR > 42.86). The reasons are that the TI showed an offset effect and ER increased the haze pollution. ER aggravated haze pollution by IS, attributed to the “Chinese decentralization” system. The high level of urbanization means high energy consumption and more pollutant emissions, and the binding effect of ER of cities with high-level urbanization is weakened. As for the EC, the relationship between ER and haze pollution changed to an inverted U-shaped. The reasons are that China’s power generation is dominated by coal, resulting in minimal environmental regulations efficiency. However, the issued policies and cleaner power generation are effective in improving the environmental efficiency of the power industry. Under the constraints of ER, haze pollution is significantly mitigated by the FDI mechanism due to the “pollution halo” effect, and this mitigation effect is increasing, indicating that the introduction of foreign investment and the rational use of associated technology will greatly reduce haze pollution.

After a comprehensive analysis, the direct and indirect threshold effects show obvious regional distribution characteristics. The regional distribution of TI, IS, and UR is similar to ER; ER will increase haze pollution after exceeding the corresponding threshold. There are fewer cities with high frequencies of FDI and EC which cannot achieve a large-scale haze reduction effect. The interaction of these indirect effects has caused a direct impact of ER on haze control.

### 5.2. Policy Implications

According to the research results, the relevant policies are as follows: (1) The Government should formulate corresponding environmental supervision policies to maximize the effectiveness of environmental regulations and prevent the expansion of the negative effects. (2) Local governments should incorporate environmental benefits into the cadre performance appraisal system while pursuing economic effects to avoid competition between local governments that affects the effectiveness of environmental regulations. (3) A knowledge transfer system will be formed, and areas with relatively developed technology will share results with less-developed areas in order to achieve balanced development. (4) At this stage, the Government should make full use of foreign direct investment to enhance the positive effects of foreign direct investment, so as to achieve pollution prevention and control, and prevent itself from becoming a “pollution heaven”. (5) Under the premise that the overall urbanization rate in China is relatively high, the Government should appropriately control the urbanization rate, strengthen the efficiency of implemented environmental regulations, and realize the national prevention and control of haze pollution. (6) Local-level cities should combine their strengths to accelerate the development of clean energy and realize clean production of electricity supply.

Although China has achieved world-renowned developmental achievements, this has been unfortunately accompanied by environmental degradation. Importantly, this deterioration will seriously affect economic development and public health. Environmental governance is urgent, and all regions should strengthen policy synergy to realize “lucid waters and lush mountains”.

## Figures and Tables

**Table 1:** The descriptive statistics of all variables.

Variable	Definition (Unit)	Type	Mean	Std. Dev.	References
PM_2.5_	PM_2.5_ concentration (ug/m^3^)	Dependent variable	37.76	15.96	[2,13]
ER	Environmental regulations (%)	Independent variable	40.41	14.47	[14,46,47]
TI	The proportion of Government expenditure for science and technology in regional GDP (%)	Transition variables	0.30	0.41	[11,48]
IS	The proportion of added value of secondary industry in regional GDP (%)		48.77	11.80	[5]
FDI	The proportion of FDI in regional GDP (%)		3.79	3.38	[2]
UR	The regional urbanization rate (%)		51.20	15.84	[41]
EC	The logarithm of regional electricity consumption (KW.h)		13.22	1.22	[29,49]
LNPD	Population density (100 people/km^2^)	Control variables	6.45	0.91	[10]
LNPGDP	GDP per capita (CNY)		10.77	0.62	[50]
PB	Bus per 10,000 people (buses/10,000 people)		8.15	6.94	[11]
RP	Road area per capita (m^2^)		12.17	9.14	[24]
KQ	Air flow coefficient (10 m^2^/s)		7.52	0.54	[14,51]

**Table 2:** The results of vocabulary statistics of government work reports in 2018.

Province	I	II	III	IV	V	VI	VII	VIII	IX	X	XI	EV	TV
Anhui	1	0	2	1	30	10	16	2	2	24	2	90	6784
Beijing	0	2	9	0	29	6	24	2	0	9	5	86	7090
Chongqing	1	0	4	0	32	15	16	2	1	9	2	82	6709
Fujian	0	0	6	0	44	8	17	3	2	16	3	99	6717
Gansu	0	0	5	1	52	12	9	1	1	20	1	102	7446
Guangdong	0	0	5	1	26	8	24	2	2	9	4	81	8520
Guangxi	0	0	4	2	43	11	8	2	2	5	2	79	7392
Guizhou	0	0	1	0	38	8	8	0	4	27	7	93	7410
Hainan	1	0	6	0	59	15	8	0	2	5	7	103	7929
Hebei	3	1	5	0	23	11	18	3	2	11	6	83	7077
Heilongjiang	2	2	3	0	24	3	5	2	0	9	3	53	5818
Henan	6	0	8	0	26	6	23	5	2	10	2	88	6942
Hubei	0	0	2	0	24	7	10	1	2	17	2	65	5297
Hunan	0	0	0	0	15	7	10	1	2	11	5	51	6953
Jiangsu	2	2	8	4	52	7	17	2	2	8	2	106	7551
Jiangxi	0	0	2	2	49	4	4	2	2	27	1	93	6792
Jilin	0	0	7	0	23	10	9	2	1	17	3	72	7041
Liaoning	1	1	2	1	31	3	7	1	1	7	1	56	6592
Inner Mongolia	0	0	2	1	23	5	13	1	2	12	0	59	4556
Ningxia	0	0	1	0	36	12	7	1	2	10	4	73	5949
Qinghai	0	0	1	2	51	7	4	0	2	30	1	98	6309
Shaanxi	0	0	0	1	27	8	13	2	0	6	1	58	5663
Shandong	2	0	5	0	26	9	11	3	1	8	0	65	7276
Shanghai	0	0	6	1	24	7	10	4	0	10	4	66	7094
Shanxi	2	1	6	2	29	8	15	2	1	7	2	75	7997
Sichuan	5	0	2	0	32	9	15	1	1	15	3	83	7221
Tianjin	2	0	2	1	23	7	12	1	2	21	7	78	5937
Xinjiang	0	0	8	0	50	17	17	1	0	11	2	106	8446
Yunnan	0	0	6	0	33	12	12	1	2	19	2	87	6814
Zhejiang	4	0	1	0	21	5	8	3	1	10	6	59	6056

Notes: The similar words were grouped and numbered. Each column in Table 2 corresponds to the sum of the frequencies of similar words in that category of each provincial government work report. COD (chemical oxygen demand) is the amount of oxygen needed to oxidize the organic matter present in water, and it is a comprehensive indicator of the concentration of reducing pollutants in wastewater. Its unit is mg/L and expressed by the abbreviation COD.

**Table 3:** Panel unit root results.

Variables	ADF-Fisher Chi-Square Statistics (*p*-Value) at Levels	ADF-Fisher Chi-Square Statistics (*p*-Value) at First Difference	LLC Statistics (*p*-Value) at Levels	LLC Statistics (*p*-Value) at First Difference
PM_2.5_	1608.64 (0.000) ***	1724.74 (0.000) ***	−17.08 (0.000) ***	−18.67 (0.000) ***
ER	1398.19 (0.000) ***	1376.15 (0.000) ***	−3.97 (0.000) ***	−7.39 (0.000) ***
TI	1561.28 (0.000) ***	1507.38 (0.000) ***	−18.97 (0.000) ***	−19.96 (0.000) ***
IS	1189.18 (0.000) ***	1139.01 (0.000) ***	−10.54 (0.000) ***	−12.61 (0.000) ***
FDI	1597.90 (0.000) ***	1500.56 (0.000) ***	−10.38 (0.000) ***	−10.24 (0.000) ***
UR	1384.07 (0.000) ***	1284.10 (0.000) ***	−23.54 (0.000) ***	−49.17 (0.000) ***
EC	1013.10 (0.000) ***	1053.37 (0.000) ***	−6.28 (0.000) ***	−8.10 (0.000) ***
LNPD	2378.82 (0.000) ***	2246.13 (0.000) ***	−31.18 (0.000) ***	−22.18 (0.000) ***
LNPGDP	1640.35 (0.000) ***	1558.08 (0.000) ***	−14.87 (0.000) ***	−13.98 (0.000) ***
RP	1316.26 (0.000) ***	1250.15 (0.000) ***	−11.54 (0.000) ***	−9.81 (0.000) ***
PB	1468.63 (0.000) ***	1314.76 (0.000) ***	−12.87 (0.000) ***	−7.47 (0.000) ***
KQ	1979.69 (0.000) ***	2016.90 (0.000) ***	−21.22 (0.000) ***	−30.32 (0.000) ***

Notes: (***), (**) and (*) denote significance at 1%, 5% and 10%, respectively. Null hypothesis: series has a unit root.

**Table 4:** Linearity test results.

Statistic	Threshold Variable					
	ER	TI	IS	FDI	UR	EC
H_0_: linear model (r = 0) vs. H_1_: PSTR model with at least one threshold variable (r = 1)						
Wald LM test (LMw)	64.03 ***	30.14 ***	38.91 ***	36.91 ***	50.74 ***	69.76 ***
	0.000	0.000	0.000	0.000	0.000	0.000
Fisher LM test (LMF)	5.38 ***	4.60 ***	5.95 ***	5.64 ***	7.80 ***	10.78 ***
	0.000	0.000	0.000	0.000	0.000	0.000
Likelihood ratio test (LRT)	64.69 ***	30.29 ***	39.16 ***	37.13 ***	51.16 ***	70.55 ***
	0.000	0.000	0.000	0.000	0.000	0.000

Notes: (***), (**) and (*) denote significance at 1%, 5% and 10%, respectively. Null hypothesis: linear model.

**Table 5:** Test of no remaining linearity: test for the number of regimes.

Statistic	Threshold Variable					
	ER	TI	IS	FDI	UR	EC
H_0_: r = 1 vs. H_1_: r = 2						
Wald LM test (LMw)	14.43	1.35	15.22	7.73	15.49	12.23
	0.21	0.96	0.19	0.25	0.11	0.15
Fisher LM test (LMF)	1.18	0.20	2.30	1.16	2.34	1.84
	0.29	0.97	0.32	0.32	0.12	0.18
Likelihood ratio test (LRT)	14.47	1.35	15.26	7.74	15.52	12.26
	0.208	0.96	0.18	0.25	0.11	0.15

Notes: (***), (**) and (*) denote significance at 1%, 5% and 10%, respectively. Null hypothesis: no remaining non-linear model.

**Table 6:** Results of panel smooth transition regression model.

Core Explanatory Variable	Interpreted Variable: PM_2.5_					
	Threshold Variables					
	ER	TI	IS	FDI	UR	EC
ER (*β*_0_)	−0.06 ***	−0.05 **	−0.001 ***	−0.02 ***	−0.16 ***	1.22 ***
	0.00	0.02	0.00	0.00	0.000	0.001
ER (*β*_1_)	0.08	0.19 ***	0.002 ***	−0.08 **	0.20 ***	−1.55 **
	0.20	0.00	0.00	0.091	0.000	0.01
*β*_0_ + *β*_1_	0.02	0.14	0.001	−0.10	0.04	−0.33
Threshold (c)	38.86	0.37	39.61	7.25	42.86	13.75
Slope (γ)	0.17	493.08	1.01	6.24	0.32	3.10

Notes: (***), (**) and (*) denote significance at 1%, 5% and 10%, respectively.

## Data Availability

The Supplementary Materials provides most of the data, the remaining data are not publicly available due to part of them are being used in other studies that have not yet been publicly published. The datasets used during the current study are available from the corresponding author upon reasonable request—Yonglian Chang.

## Supplementary Materials

The following are available online at https://www.mdpi.com/article/10.3390/ijerph182312423/s1, Supplementary S1: The Matlab code of the PSTR.
